# Artificial intelligence in cardiology: Hope for the future and power for the present

**DOI:** 10.3389/fcvm.2022.945726

**Published:** 2022-10-13

**Authors:** Loucia Karatzia, Nay Aung, Dunja Aksentijevic

**Affiliations:** ^1^Centre for Biochemical Pharmacology, William Harvey Research Institute, Barts and The London School of Medicine and Dentistry, Queen Mary University of London, London, United Kingdom; ^2^Centre for Advanced Cardiovascular Imaging, William Harvey Research Institute, Barts and The London School of Medicine and Dentistry, Queen Mary University of London, London, United Kingdom; ^3^National Institute for Health and Care Research (NIHR) Barts Biomedical Research Centre, William Harvey Research Institute, Barts and The London School of Medicine and Dentistry, Queen Mary University of London, London, United Kingdom

**Keywords:** artificial intelligence, cardiology, machine learning, cardiac imaging, cardiac MR (CMR), cardiovascular diagnostic

## Abstract

Cardiovascular disease (CVD) is the principal cause of mortality and morbidity globally. With the pressures for improved care and translation of the latest medical advances and knowledge to an actionable plan, clinical decision-making for cardiologists is challenging. Artificial Intelligence (AI) is a field in computer science that studies the design of intelligent agents which take the best feasible action in a situation. It incorporates the use of computational algorithms which simulate and perform tasks that traditionally require human intelligence such as problem solving and learning. Whilst medicine is arguably the last to apply AI in its everyday routine, cardiology is at the forefront of AI revolution in the medical field. The development of AI methods for accurate prediction of CVD outcomes, non-invasive diagnosis of coronary artery disease (CAD), detection of malignant arrythmias through wearables, and diagnosis, treatment strategies and prediction of outcomes for heart failure (HF) patients, demonstrates the potential of AI in future cardiology. With the advancements of AI, Internet of Things (IoT) and the promotion of precision medicine, the future of cardiology will be heavily based on these innovative digital technologies. Despite this, ethical dilemmas regarding the implementation of AI technologies in real-world are still unaddressed.

## Highlights

- Artificial intelligence is a computer science field that studies the problem of building agents which take the best possible course of action in a specific situation.
- Cardiology is at the forefront of artificial intelligence revolution in medicine.

- AI allows accurate prediction of cardiovascular outcomes, non-invasive diagnosis of coronary artery disease, detection of malignant arrhythmias and diagnosis, treatment, and prediction of outcomes for heart failure patients.
- The advancements of artificial intelligence, Internet of Things, and precision medicine will lead to future innovations in the field of cardiovascular research.
- Artificial intelligence in cardiology is limited by ethical and data privacy concerns, which are still to be addressed.
- Regulations are required to be implemented for the safe use of artificial intelligence in cardiology and medicine in the future.

## Introduction

Cardiovascular disease (CVD) is the principal cause of mortality and morbidity globally. The diagnosis and treatment of CVD relies on data in several forms, such as patient history, physical examination, laboratory data, invasive and non-invasive imaging techniques. With the pressures for improved care and translation of the latest medical advances and knowledge to an actionable plan, clinical decision-making for cardiologists is challenging (1). On the other hand, emerging new technologies and the growth of artificial intelligence (AI) and machine learning (ML) in the last few decades have offered physicians opportunities to conduct more efficient and data-driven research. The availability of large-volume data from electronic health records (EHRs), mobile health devices and imaging data enables the rapid development of AI algorithms in medicine. Cardiology has been one of the few medical specialties in which AI technologies have been examined systematically (2).

## Artificial intelligence

It is difficult to determine the exact year that AI was born. The English mathematician Alan Turing, named by some as the father of AI, developed the famous code breaking machine *The Bombe* for the British government, which broke the Enigma code, used by the German army in the Second World War. The Bombe was considered the first working electro-mechanical computer (3). In 1950, inspired by his achievement, Turing published his article “*Computing Machinery and Intelligence*,” where he proposed the famous question “*Can machines think?*” and recommended definitions for the terms machine and think (4). He also outlined the world known Turing Test–which is considered today as the standard method to identify intelligence of an artificial system. According to the Turing Test, if a human is interacting with another human and a machine and cannot distinguish the machine from the human, then the machine is considered to be intelligent (3). In 1955, during the Dartmouth Research Project, AI was defined as the problem of “*making a machine behave in ways that would be called intelligent if a human were so behaving*” (5). In 1968, the cognitive scientist Marvin Minsky outlined AI as the “*science of making machines do things that would require intelligence if one by men*” (6). More recently, in his published paper “*What is Artificial Intelligence*,” John McCarthy gives the following explanation regarding AI: “*It is the science and engineering of making intelligent machines, especially intelligent computer programs. It is related to the similar task of using computers to understand human intelligence, but AI does not have to confine itself to methods that are biologically observable*” (7). Kaplan and Haenlein in 2019 summarised AI as “*a system’s ability to interpret data correctly, to learn from such data, and to use those learnings to achieve specific goals and tasks through flexible learning*” (3). Stuart Russell and Peter Norvig, authors of “*Artificial Intelligence: A Modern Approach*,” have defined AI by dividing it into four goal-based categories (Table 1; 8). The definitions are laid out in two scopes. One dimension is whether the goal of AI is to match human performance or ideal rationality. The other aspect is whether the goal is to build systems that think or systems that act (9).

**TABLE 1:** Definitions of Artificial Intelligence (AI) by four goal based categories (8).

Acting humanly	Acting rationally
“The art of creating machines that perform functions that require intelligence when performed by people.” (107)	“Computational intelligence is the study of the design of intelligent agents.” (108)

**Thinking humanly**	**Thinking rationally**

“The exciting new effort to make computers think… *machines with minds*, in the full and literal sense.” (109)	“The study of mental faculties through the use of computational models.” (110)

Traditionally, statistics has been the standard method used in medical research to show the benefit of new treatments, identify risk factors for a disease and predict prognosis. Traditional medical research proposes a hypothesis, which is then tested with statistical analysis. Statistics analyses a given dataset using mathematical equations and discovers relationships between data points and outcomes. It is focused on validation of the hypothesis and understating of the causality and the mechanisms (10). AI is data driven and does not require the formulation of a hypothesis. It makes predictions with high accuracy, without needing to interpret the data given (1). Its goal is to identify hidden patterns in the data and predict new data. AI is able to use very complex nonparametric models from a vast amount of data in comparison to simple parametric models requiring a suitable-sized data set used in statistics (10).

### Machine learning

**FIGURE 1:**
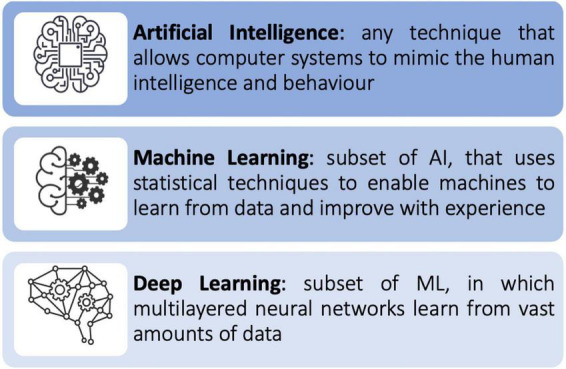
Relationship between Artificial Intelligence (AI), Machine Learning (ML), and Deep Learning (DL).

**FIGURE 2:**
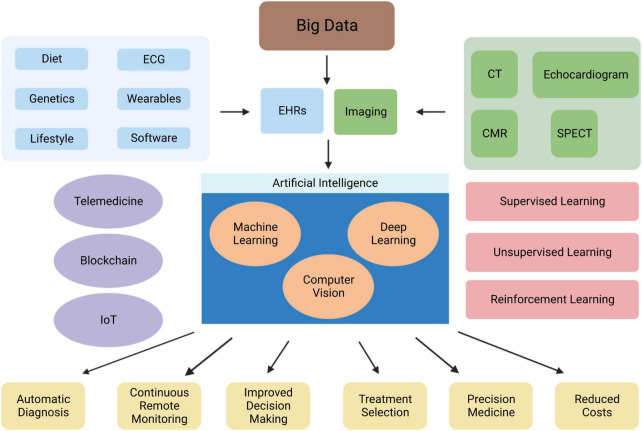
Overview of the role of AI in cardiovascular medicine. Abbreviations: EHRs, electronic health records; CMR, cardiac magnetic resonance; CT, computed tomography; IoT, internet of things; SPECT, single photon-emission computerised tomography.

Machine learning (ML) is a subfield of AI (Figure 1). ML allows a system to learn from data rather than through explicit programming. It uses algorithms that learn from data, identify specific patterns, and make decisions/predict outcomes based on the learned model (10). Once the ML algorithm is trained with data, the ML model will be provided with an input. The output will be a predictive model, based on the data that trained the model (11). In ML, the machine learns from the data through three different methods: supervised, unsupervised, and reinforcement learning (Figure 2).

Supervised learning is the most widely used technique today. The model is provided both the inputs and the outputs at the time of training. It then goes on to produce a prediction model through categorising future events or finding which variables are most applicable to the outcome (1). Most commonly used supervised learning tasks include classification (identification of the group a new measurement belongs to) and regression (prediction of a continuous value of a new observation) (10).

In unsupervised learning the model learns patterns from the input, without feedback provided from humans (8). Therefore, the algorithm needs to explore the data and find hidden patterns. The most common tasks in unsupervised learning are clustering (dividing objects in groups with similar characteristics) and dimensionality reduction (reducing the number of variables of data through keeping initial variables that explain the data), which can also be applied in supervised learning (10).

Reinforcement learning involves receiving an output variable to be amplified and a sequence of choices that can be taken in order to influence the output (3). The model is not told which actions to take, but it needs to discover which actions will lead to the highest level of reward, by trying them. Trial-and-error-search and delayed-reward are the two features that distinguish reinforcement learning (12).

Machine learning operates *via* a variety of algorithms, which serve different tasks. Understanding the different types of ML algorithms aids the researcher to choose the best type of algorithm for their project. One of the most known examples is the *Bayesian algorithm*. It allows the scientist to encode previous beliefs of what a model should look like, irrespectively of what the data states. This is used in cases where the amount of data available is small for the purpose of training a model. *Decision tree algorithms* use a branching like structure to map possible outcomes of a decision, with a percentage assigned to each node, depending on the chance of the outcome occurring (11). A *random forest algorithm* consists of the output of multiple decision trees, to reach a single result. *Support vector machine algorithms* draw a boundary line which increases the margins from each class and new observations are classified based on this line (10). *Ensemble learning* is another ML method, in which multiple weak algorithms are combined to obtain a good prediction. Bagging, boosting, and stacking are the three approaches of ensemble learning. In bagging, multiple weak learners (algorithms) are trained in parallel, and the results of each learner are combined to produce a final output. In boosting, multiple weak learners are combined in series and trained consequently, considering errors from previous algorithms, in order to reduce bias. In stacking, the results of weak learners are used as input for another ML algorithm (10).

### Deep learning

Deep learning (DL) is a subfield of ML (Figure 1). It is inspired by the complexity of the human brain in handling data and generating patterns, used for decision making (1). DL is comprised of deep neural networks. A neural network has three or more layers: an input layer, one or many hidden layers, and an output layer. Data is consumed *via* the input layer. Then the hidden layers extract the salient features from the input data to produce an output that closely approximates the ground-truth. A neural network may comprise of millions of simple processing nodes which are firmly unified (11). In DL there are multiple hidden tiers of artificial neural networks that can create automated forecasts from training datasets. DL requires complex data for training but is not required to extract features from the input data. Once implemented it is self-directed, thus eliminating manual human interaction. It can extract important results from vast amount of data through iterative processing (10).

Neural networks can be used for various tasks such as classification, clustering, dimensionality reduction, pattern recognition, natural language processing, computer vision, and predictive analysis (13). Neural networks consist of multiple layers of interconnected artificial neurons. A neuron receives inputs multiplied with random weights, to which a bias value is then added. An activation function is then applied and defines the final value to be given out of the neuron. There are different types of activation functions, depending on the input values (14). Neural networks are classified depending on their structure, data flow, neurons used and their density. The most important types of neural networks involve:

1. Feed Forward Neural Network
2. Multilayer Perceptron
3. Radial Basis Function Neural Network
4. Recurrent Neural Network
5. Modular Neural Network
6. Convolutional Neural Network

*Feed Forward Neural Networks (FFNNs)* are the simplest form of neural networks, as data travels in just one direction, passing from input and exiting through output nodes (hidden layers may or may not be present). They can be either single-layered or multi-layered FFNNs and the number of layers depend on how complex the function is. FFNNs are usually applied in face recognition or simple classification. In *Multiplayer Perceptron (MLP)*, input data travels through various layers of artificial neurons. It is a fully connected neural network, as all nodes are connected to all the neurons in the next layer. Input and output layers and multiple hidden layers (three or more) are present, and propagation is bi-directional (forward and backward). MLP is used in speech recognition, machine translation and complex classification. In a *Recurrent Neural Network* (RNN), the output of a layer becomes the input to the next layer—which is the only layer in the network. Therefore, the output of a layer becomes an input to itself and forms a feedback loop. This means then network has internal memory, which influences the current output. RNNs are used in tasks such as text processing and text to speech processing. An example of this is when typing in Google, it automatically completes the sentence for us! The *Radial Basis Function Network* (RBFN) is based on the radial basis function (activation function), which is included in the hidden layer. The input is designated to a centre and the output combines the outputs of the radial basis function and weight parameters to perform classification or inference. It is used for prediction analysis and function approximation. A *Modular Neural Network* (MNN) is consisted of multiple different networks which work independently and perform different tasks, towards achieving the output. It is usually used in stock market prediction systems and in cases of compression of high level input data (13).

### Convolutional neural networks

**FIGURE 3:**
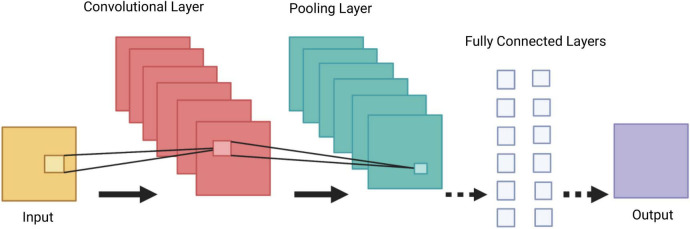
Demonstration of a Convolutional Neural Network (CNN) architecture. A CNN is composed of several blocks which include convolutional layers, pooling layers, and fully connected layers.

*Convolutional Neural Networks* (CNNs) are a group of deep neural networks, used in various fields including face recognition, speech processing and computer vision. They are a powerful tool in DL, as they necessitate minimal amount of pre-processing information (15). The CNN’s architecture is inspired by neurons in human and animal brains. It consists of multiple stacks (blocks) of convolution layers and pooling layers, followed by a fully connected layer and a normalising layer (Figure 3). The convolutional layer is the most important component of the CNN architecture. Its convolutional topology allows CNNs to perform dimensionality reduction, effective automated feature extraction (in contrast to traditional algorithms’ labour hand-crafted feature extraction) and perform operations from 2D and 3D images. Its most important characteristics, *weight sharing* (all neurons of neighbouring layers share the same weight) and *local connectivity* [neurons in one layer are connected to neurons in the next layer that are spatially close to them, thus keeping the ones that carry the most important information (memory-effective)], make the CNN’s training process more simplified and efficient, as a small number of parameters is utilised with minimal human effort (16).

A collection of filters (kernels), which are part of the convolutional layer, perform convolution (a sliding window across an input, creating one averaged output for each stride the window takes) of the input image to generate output feature maps. The outputs are passed to an activation function. The most common non-linear activation function currently used for CNNs is ReLU. The large feature maps created by convolutional operations, are then sub-sampled by the pooling layers. The basic idea of down-sampling is that only the most important information of the feature map is maintained. In the fully connected layer, each neuron is connected to all neurons of the previous layer, thus forming a fully connected neural network. When the features extracted by the convolution layers and sub-sampled by the pooling layers are created, they are mapped by fully connected layers to the final outputs of the network, same as the probability for each class in classification tasks. An activation function is also applied to the last fully connected layer, depending on the task. For multiclass classification task, the softmax function converts the output real values from the last fully connected layer to aim class probabilities, ranging from 0 to 1 and with a sum of 1 (17). Multiple different CNN architectures have been created over the last few years. Model architecture is vital in performance improvement of different applications (Table 2).

**TABLE 2:** Most used Convolutional Neural Network (CNN) architectures.

Model	Main task	Dataset	Error rate	Input size	Year
AlexNet	Uses Dropout and ReLu	ImageNet	16.4	227 × 227 × 3	2012
VGG	Small filter size, increased depth	ImageNet	7.3	224 × 224 × 3	2014
GoogleNet	Different filter size, increased depth, block concept, concatenation concept	ImageNet	6.7	224 × 224 × 3	2015
ResNet	Robust against overfitting due to symmetry mapping-based skip links	ImageNet	3.57	224 × 224 × 3	2016
DenseNet	Blocks of layers—layers connected to each other	CIFAR-10, CIFAR100, ImageNet	3.46, 17.18, 5.54	224 × 224 × 3	2017

The aim of a CNN’s training is to find kernels (filters) in convolution layers and weights in fully connected layers which will reduce the difference between output predictions and ground truth labels on a training dataset. The data available is split into a training set, a validation set and a test set. The training set is used to train the network, in two phases. In forward propagation, the input is passed completely through the network, where loss values are calculated. In backpropagation, each layer will receive the gradient of loss with respect to its outputs and return the gradient of loss with respect to its inputs, leading to update of the learnable parameters. The validation set evaluates the model during the training process and performs model selection. The test set is used at the end to evaluate the performance of the final model selected in the previous steps (17). The CNN’s architecture and function, makes it attractive for the purpose of solving classification problems in a high dimensional space. The reason for this is that CNNs are universal, meaning they can be used to approximate any continuous function to an arbitrary accuracy when the depth of the neural network is large enough. CNNs and DL in general, work well in exceptionally large networks with a vast number of parameters, as they are surprisingly good at extrapolating when fed data similar to what they were trained on (18).

### Generative adversarial networks

Generative adversarial networks (GANs) were introduced by Goodfellow et al. in, as a new framework for the creation of synthetic data, which aim to mimic the real dataset (19). GANs are an unsupervised learning algorithm and consist of two neural networks. The generator network generates new examples, and the discriminator network evaluates whether the generated examples belong to the real training dataset (classifies them as real or fake). The two models are trained at the same time, until the generator is generating realistic examples. GANs have been mainly used to generate images and healthcare records data in the medical field (20). For example, Amirrajab et al. used a heart stimulator and combined it with a GAN to generate synthetic short-axis cine CMR images at multiple slice locations (21). In a different study, a GAN was used to convert CMR images to computed tomography, for better visualisation of calcified structures, which are difficult to detect on CMR (22). GANs have shown to improve the performance of predictive models by filling the missing data. Che et al., added GAN-generated synthetic data to real patient data, leading to an improved CNN-based risk prediction model (23).

### Limitations of artificial intelligence models

Machine learning models are best trained and have higher accuracy when using big data. Big data is any kind of data source that has the following characteristics: exceptionally large amount of data, the capability to transfer that data at a great velocity of speed, a mounting range of data sources and validity so that data sources reflect the truth (11). A large dataset allows for subsampling of the data for bootstrapping approaches (thus providing measures of robustness of an approach) and computational reasons (a model structure can be developed on a subset of a large dataset). In the case of a small DL dataset, techniques such as data augmentation (modification of training data through random transformations so the model does not see the same inputs during the training iterations) and transfer learning (features learned on a large dataset can be shared in a similar target dataset) can be applied, to train the model efficiently.

Overfitting is one of the most important issues that need to be addressed when building an AI model. Effectively overfitting violates the principle of parsimony, a well-known law in statistical analysis. Parsimony highlights that a problem should be stated in the simplest possible terms and explained with the fewest assumptions possible. With overfitting, the model tries to fit the training data entirely and ends up memorising irrelevant data patterns, noise and random fluctuations and performs less well in a subsequent unseen dataset. This can be recognised in cases where the model performs well on the training set but not on the validation set. Cross-validation can detect overfitting by identifying how well the model can generalise to other datasets. Regular monitoring of the loss and accuracy on the training and validation sets, can lead to early recognition of overfitting. Obtaining a larger training dataset, data augmentation and reduction of architectural complexity are some of the ways overfitting can be mitigated (17).

Outliers are another important issue in AI applications. These are values that look different (are in the extremes) from other values in the data, may carry undue weight on their final classification and therefore mislead the training process and produce less accurate models. The ML algorithm should be able to deal with outliers and similar technical problems if challenged. Detection of outliers through visualisation techniques (i.e., box plot) and mathematical functions and prevention of them *via* larger datasets, can solve this problem (24).

When dealing with large datasets, it is important to be aware of the risks when calculating the effect size and the statistical significance. Effect size is essentially the quantification of the size of difference between two groups. Statistical significance provides the likelihood that the difference between two groups could be an accident of sampling and is calculated with the *p*-value. The main issue of statistical significance is that the *p*-value depends on the size of the effect and the size of the sample. With larger datasets, everything becomes statistically significant, even if practically is not significant. Less emphasis should be given on *p*-values and more importance should be attributed to the effect size calculation along with a margin error/confidence interval when involving big data, as larger datasets produce more accurate effect size (25).

The widespread use of Big Data in the field of AI, does not come without challenges. As a dataset grows larger, it can lead to class imbalance. The ML model’s performance decreases, as datasets include data from classes with various probabilities of occurrence (26). Different methods have been applied to solve this issue, including down-sampling large classes or up-sampling small classes, and constructing models for every hierarchical level (17). As the volume of data increases, variance and bias also increases. Variance involves the consistency of a learner’s ability to predict random things, whilst bias is the learner’s ability to learn the wrong thing. If bias is introduced in the data, generalisation of the data is compromised. Regularisation techniques are well-established methods in ML which improve generalisation (26).

Adversarial attacks compromise the reliability and robustness of DL methods and their safe application in medicine. They encompass mildly altered images, which resemble original images, but they are maliciously designed to confuse pre-trained models. This can lead to a completely different prediction for the image the neural network analyses. Various methods are being proposed for defence against such attacks, but none has been proven safe enough yet (27). Lastly, what a neural network considers meaningful information for extraction from the data presented to it, remains an unaddressed question. Attention mapping is a scalar matrix, which aims to augment the significant image regions and suppress the irrelevant information in other regions, with respect to the target task. It amplifies the importance of input variables in terms of their impact on outcomes (28). Whilst attention mechanisms are potentially able to boost the performance of a neural network, they are not without limitations. They require vast amount of data for training, are not robust when generalised in other tasks other than the one they were trained for, cannot control spurious correlations in the data and no research has been undertaken to compare different attention models’ performances (29, 30).

## Applications of artificial intelligence in cardiology

Artificial intelligence applications in cardiovascular research are increasingly becoming more popular over the last decade (Figure 2). AI algorithms have been broadly used for diagnosis from an image, image segmentation and reconstruction, image quality control, patient prognostication, phenogrouping, and gaining of scientific insight. Patient meta-data (demographics, co-morbidity) has been used to improve the performance of ML algorithms. AI based software devices and risk assessment tools have also been adapted in the field of Cardiology. Table 3 demonstrates examples of such achievements.

**TABLE 3:** Overview of the use of AI in cardiology.

Application/Task	Model	Data	Training set	Testing set	Accuracy/specificity/ sensitivity	References
Identification of arrythmia (SR, LBBB, RBBB, PVC, PAC)	SVM	ECG	-	-	100% (SR), 98.66% (LBBB), 100% (RBBB), 99.66% (PVC), and 100% (PAC) accuracy	(15)
Discrimination of HCM from ATH	Ensemble ML (SVM, RF, ANNs)	Clinical, echocardiography	-	-	87% sensitivity and 82% specificity	(32)
Prediction of ACM in patients with suspected CAD undergoing CTA	Boosted ensemble algorithm	Clinical, CTA	-	10,030 subjects	AUC 0.79	(40)
Arrythmia detection (prediction of 12 types of arrythmia compared to cardiologist)	DNN	ECG	-	-	99% accuracy	(13)
Detection of subclinical AF	CNN	ECG	454,789 images	130,801 images	AUC 0.90, sensitivity 82.3%, specificity 83.4%, accuracy 83.3%	(17)
Identification of ventricular dysfunction (EF 35%)	CNN	ECG, echocardiography	44,959 subjects	52,870 subjects	AUC 0.93, sensitivity 86.3%, specificity 85.7%, and accuracy 85.7%	(52)
Phenogroup HF patients and identification of responders to CRT implantation	Unsupervised ML (Multiple Kernel Learning and K-means clustering)	Clinical, echocardiography	-	1,106 subjects	-	(60)
Automated analysis of cardiac structure and function (left ventricular chamber volumes, mass and EF)	CNN	CMR	599 subjects	110 subjects	-	(47)
Prediction of CAD on CTA	Boosted ensemble algorithm	Clinical, CTA (CACS)	-	13,054 subjects	AUC 0.881	(38)
Prediction of ACM for 1, 2-, 3-, 4-, and 5-years post CRT implantation	RF	Clinical, ECG, echocardiography	2,282 subjects	1,510 subjects	AUC 0.768 (1 year), 0.793 (2 years), 0.785 (3 years), 0.776 (4 years), 0.803 (5 years)	(59)
Prediction of early coronary revascularisation within 90 days after SPECT MPI	Ensemble LogitBoost algorithm	Clinical, SPECT	-	1,980 subjects	AUC 0.81	(44)
Identification of patients with PAH	Tensor based ML algorithm (multilinear subspace learning)	CMR	200 subjects	1,122 subjects	AUC 0.92	(48)

ACM, all-cause mortality; AF, atrial fibrillation; ANNs, artificial neural networks, ATH, athlete’s heart; AUC, area under the curve [integral of the ROC (receiver operator characteristic) curve]; CACS, coronary artery calcium score; CAD, coronary artery disease; CMR, cardiovascular magnetic resonance imaging; CNN, convolutional neural network; CRT, cardiac resynchronisation therapy; CTA, Cardiac Computed Tomography Angiography; DNN, deep neural network; ECG, electrocardiogram; EF, ejection fraction; HCM, hypertrophic cardiomyopathy; HF, heart failure; LBBB, left bundle branch block; ML, machine learning; MPI, myocardial perfusion imaging; PAC, premature atrial contraction; PAH, pulmonary arterial hypertension; PVC, premature ventricular contraction; RBBB, right bundle branch block; RF, random forest; SPECT, single-proton emission computerised tomography; SR, sinus rhythm; SVM, support vector machine.

### Electrocardiography

The electrocardiogram (ECG) is considered the first-line non-invasive diagnostic investigation for the evaluation of cardiovascular pathology. However, its interpretation can be time-consuming and challenging at times. Automated ECG interpretation, *via* digital ECG machines nowadays is almost universal. Despite the significant progress made in the development of computerised interpretation of the ECG, various limitations are still present, with systematic over-reading of the ECG deemed necessary (31). AI methods are increasingly used with aim to improve the accuracy of automated ECG interpretation and aid patient stratification and prognostication. Modern ML models identify the P and T waves and the QRS complexes and calculate parameters such as the heart rate (HR), the cardiac axis, different interval lengths of a patient’s ECG, ST-changes and common rhythm abnormalities such as atrial fibrillation (AF) (32). In a recent study, a 34-layer DNN (33 convolutional layers followed by a linear output layer into a softmax) has been developed, which detects various arrythmias and outperforms a board-certified cardiologist in recall and precision (33). In another study, three neural networks—back propagation, self-organising map and radial basis function—were used to categorise ECG indicators of cardiac patients into three states (normal, abnormal, and life-threatening) and were found to be accurate in 99% of test cases (34). Zhao et al., used a support vector machine (SVM) and identified five common arrythmias from ECG tracings of a large dataset. Sinus rhythm (SR), left bundle branch block (LBBB), right bundle branch block (RBBB), premature ventricular contraction (PVC), and premature atrial contraction (PAC) were classified with accuracy of 100, 98.66, 100, 99.66, and 100, respectively (35).

Atrial fibrillation is one of the most common arrythmias. Subclinical AF can cause strokes, which can lead to disability and premature death. Efforts to diagnose asymptomatic AF are increasingly gaining momentum. Recently, a risk prediction model (baseline and time-varying neural networks) was used for AF diagnosis from a cohort of 604,135 patients in a retrospective study. At the end of the follow up period (8 years), 3% of subjects had been diagnosed with AF, with the algorithm achieving an area under the curve (AUC) [the integral of the ROC (receiver operator characteristic) curve, thus the proportion of correctly classified outcomes] of 0.87 in differentiating between patients with AF and those without AF (36). In a different study, CNNs were used to screen 12-lead ECGs for features not noticeable by the physician and detected subclinical paroxysmal AF from ECGs with normal rhythm (SR). 454,789 ECGs from 126,526 individuals were included in the training dataset, 64,340 ECGs were included in the internal validation set and 130,802 ECGs in the testing set (37).

The decision to start antithrombotic therapy for patients with newly diagnosed AF relies on the balance between two risk stratification methods. The CHA2DS2-VASc score measures the possibility of a future ischemic stroke and the HAS-BLED score predicts a patient’s bleeding risk. They both aid in the decision-making process of AF treatment on an individual basis. In a recent retrospective cohort study of 9,670 patients, diagnosed with non-valvular AF and followed up for up to 1 year, multilabel ML methods were compared to the currently used risk scores for prediction of outcomes in AF patients. SVM, gradient boosting machine (GBM) and multi-layer neural networks (MLNN) were the ML algorithms used to predict a patient’s risk of ischemic stroke, major bleeding and death, and were compared to clinical risk scores by the AUC. GBM, the best performing ML algorithm of all, showed modest performance improvement for stroke compared to CHA2DS2-VASc (AUC = 0.685 vs. AUC = 0.652), but significant improvement in predicting major bleeding in comparison to HAS-BLED (AUC = 0.709 vs. AUC = 0.522) and death in comparison to CHA2DS2-VASc (AUC = 0.765 vs. AUC = 0.606) (38).

### Artificial intelligence and machine learning medical devices

Atrial fibrillation detection can be a difficult task as the current diagnostic methods (pulse palpation, ECG, ambulatory Holter monitoring) all have limitations. Today various mobile devices can be used for detection of AF. These include smartphones, smart bands or smartwatches, earlobe sensors, and handheld electrocardiogram devices. These devices are characterised by their non-invasive nature, safety, and instantaneous access to patients (39).

Wearable devices are user friendly and allow uninterrupted monitoring and instantaneous individual analysis of ECG signals. The Apple Watch and AliveCor are the most distinguished examples of wearable. The KardiaBand from AliverCor is an example of a smartphone application, based on ML for the recognition of AF from an ECG (40). In a RCT of AF screening, the AliveCor Kardia monitor connected to a WiFi-enabled iPod attained iECGs from 1,001 ambulatory patients. The study showed that screening with a twice weekly single lead iECG and remote analysis in ambulant patients aged 65 and above at high risk of stroke, was considerably more likely to detect AF in comparison to routine monitoring over a 12-month period (41). In another RCT the occurrence of recurrent AF or atrial flutter *via* daily ECG self-recordings and the time to treatment of the recurring arrythmia in patients undertaking catheter radiofrequency ablation or direct current cardioversion for AF or atrial flutter, were evaluated. The chance of recurrence identification was higher in the group which used the AliveCor KardiaMobile ECG monitor (intervention group). The time from detection to treatment was also shorter for that group (42).

The Apple Heart Study showed that the utilisation of smartphones was effective in identifying patients with subclinical paroxysmal AF. It included data from 420,000 participants, with a median follow up time of 117 days. It detected 0.5% of patients with possibly irregular pulse, 34% of which were diagnosed with AF confirmed by ECG. The notification group (set informed of irregular pulse) had higher possibility of commencing anticoagulant or antiplatelet treatment. Also, from the patients diagnosed with AF, 24% underwent cardioversion, 3% received an implantable loop recorder, 20% started anti-arrhythmic medication, and 18% undertook ablation (43).

Voice technology has been increasingly utilised for mainstream use *via* voice assistants, such as Amazon’s Alexa or Google Assistant. These advanced software architectures are based on neural network techniques undertaking the task of speech recognition. They interpret a complex conversation and generate human like responses. They are available either on smart speakers or on smartphones. Voice assistants are emerging tools for remote monitoring and undertaking of medical services. For example, they can be used for educational purposes, such as in the case of the Mayo Clinic First Aid skill, a voice application which can provide various medical guidelines including on cardio-pulmonary resuscitation. The CardioCube voice application enhances paperless medical history taking, in an outpatient cardiology clinic in Los Angeles. Patients answer verbally a set of pre-prepared questions and produce high accuracy reports in the her system. Another application by CardioCube (FCNcare), was implemented at a family care network in Belligham (USA) and allowed HF and diabetic patients to update their medical status observations from home. Telemedicine nurses obtained instant reports from the produced EHRs and were able to triage the patients accordingly. Voice applications can therefore be used as digital screening tools and red-flagging systems for patients with chronic diseases (44).

Artificial intelligence and ML based medical devices undergo a premarket review by the Food and Drug Administration (FDA), before their widespread commercial use. The FDA has three levels of clearance for AI/ML based algorithms. *510(k) clearance* is granted when the algorithm has been shown to be at least as effective as another similar legally marketed algorithm. *Premarket approval* is granted for Class III medical devices, which may have a vast impact in human health and require more thorough evaluation, to define their safety. De novo pathway approval involves those novel medical devices for which there is no similar legally marketed product but have shown to be safe and effective for use. Table 4 illustrates examples of FDA approved medical devices used in the field of cardiovascular medicine (45).

**TABLE 4:** FDA approved AI/ML based medical technologies/software.

Device name	Parent company name	Description	FDA approval number	Type of FDA approval	Date
Atrial fibrillation history feature	Apple Inc.	Detection of atrial fibrillation	K213971	510(k) premarket notification	03/06/2022
LINQ II Insertable Cardiac Monitor, Zelda AI ECG Classification System	Medtronic, Inc.	Arrhythmia detector and alarm (including ST-segment measurement and alarm)	K210484	510(k) premarket notification	11/06/2021
Gili Pro Biosensor (Also Known as Gili Biosensor System)	Continuse Biometrics Ltd.	Hardware and software for optical camera-based measurement of pulse rate, heart rate, breathing rate, and/or respiratory rat	DEN200038	*De novo* pathway	01/04/2021
Analytic for Hemodynamic Instability (AHI)	Fifth Eye Inc.	Adjunctive hemodynamic indicator with decision point	DEN200022	*De novo* pathway	01/03/2021
ECG 2.0 App	Apple Inc.	Ambulatory ECG rhythm assessment	K201525	510(k) premarket notification	08/10/2020
Bodyguard Remote Monitoring System	Preventice Technologies, Inc.	Arrhythmia detector and alarm (Including ST-segment measurement and alarm)	K192732	510(k) premarket notification	26/03/2020
AI-ECG Tracker	Shenzhen Carewell Electronics Co., Ltd	Assessment of arrhythmias using ECG data acquired from adults without pacemakers	K200036	510(k) premarket notification	20/03/2020
Eko Analysis Software	Eko Devices Inc.	Support in the evaluation of patients’ heart sounds and ECG’s	K192004	510(k) premarket notification	15/01/2020
FFRangio	CathWorks Ltd.	Analysis of previously acquired angiography DICOM data for patients with coronary artery disease	K192442	510(k) premarket notification	09/12/2019
EchoGo Core	Ultromics Ltd.	Quantification and reporting of results of cardiovascular function	K191171	510(k) premarket notification	13/11/2019
AI-Rad Companion (Cardiovascular)	Siemens Medical Solutions USA, Inc.	Image processing software for analysis from CT images to support physicians in evaluation and assessment of cardiovascular diseases	K183268	510(k) premarket notification	10/09/2019
EMurmur ID	CSD Labs GmbH	Detection and identification of heart murmurs	K181988	510(Kk) premarket notification	17/04/2019
KardiaAI	AliveCor, Inc.	Ambulatory ECG rhythm assessment	K181823	510(k) premarket notification	11/03/2019
EchoMD Automated Ejection Fraction Software	Bay Labs, Inc.	Provides automated estimation of left ventricular ejection faction based on acquired transthoracic cardiac ultrasound images	K173780	510(k) premarket notification	14/06/2018
Acumen Hypotension Prediction Index (HPI) Feature Software	Edwards Lifesciences LLC	Detection support for future hypotensive events	DEN160044	*De novo*	16/03/2018
Arterys Cardio DL	Arterys Inc.	Analysis of cardiovascular images acquired from MR scanners	K163253	510(k) premarket notification	05/01/2017
Steth IO	Stratoscientific, Inc.	Detection and amplification of sounds from the heart and lungs	K160016		15/07/2016

ECG, electrocardiogram; CT, computed tomography; MR, magnetic resonance.

### Transthoracic echocardiography

Echocardiogram is characterised by its easy application and widespread availability. It is an imaging modality that provides a real-time imaging of the heart and instant identification of any structural defects. AI enables the enhancement of imaging measurements’ accuracy, *via* the decrease in inter- and intra-operator inconsistency and by offering further information, subtle to be detected by the human eye (46). ML algorithms have been broadly used in the field of transthoracic echocardiography, with aim of diagnosis from an image, image segmentation and patient prognostication.

An innovative 2-dimensional echocardiographic image analysis system used AI-learned pattern recognition and automatically calculated left ventricular EF (LVEF) (measure of contractile function). The results of the study were similar to the results from the standard manual estimation (biplane Simpson’s method) and had less variability than visual EF (47). A multicentre study investigated the possibility of a fully automated computer vision software (AutoLV) using ML-enabled image analysis, for measurements of left ventricular volumes and EF, and average biplane longitudinal strain (LS) (a technique for evaluation of the left ventricular function). The automated measurements were achievable in 98% of the studies, with an average analysis time of 8± s per patient. The study demonstrated a rapid and efficient way of assessment LVEF and LS (48).

Another ground-breaking publication concerns the fully automated echocardiogram interpretation in clinical practice, *via* ML methods. 14,035 echocardiograms with 70,000 pre-processed images were used to train and evaluate a CNN and achieve detection of 23 viewpoints and segmentation of the cardiac chambers across 5 common views. The CNN was a VGG network which took a fixed-sized input of grayscale images, passed it through 10 convolution layers, five max-pool layers, and three fully connected layers. The output was fed into a 23-way softmax layer, to represent 23 different echocardiography views. Training data comprised of 10 random frames from each manually labelled echocardiographic video. The study found that the automated measurements were comparable or superior to the manual measurements across 11 internal consistency metrics (49). In another study, a CNN (consisting of six convolutional layers, two fully connected layers, and a softmax classifier) was trained and validated on 200,000 images, and was able to classify 15 standard echocardiography views, based on labelled still images and videos. The model classified among 12 video views with a 97.8% overall test accuracy. It also exceeded board-certified echocardiographers’ accuracy among 15 views on single low-resolution images (91.7 vs. 70.2–84, respectively) (50).

From a cardiomyopathy point of view, ML algorithms were trained from clinical, conventional echocardiography data and speckle tracking echocardiography variables, to distinguish constrictive pericarditis from restrictive cardiomyopathy. The associative memory classifier (AMC) was found to be the best performing algorithm with AUC of 89.2%. This method was found to be superior to the use of commonly used echocardiography variables, for the differentiation between these two diseases which carry many similarities (51).

Similarly, an ensemble ML algorithm model encompassed by three algorithms (SVM, RF, and MLP with back propagation), incorporated speckle-tracking echocardiographic data, to automatically distinguish the condition of inherited hypertrophic cardiomyopathy (HCM) from hypertrophy physiologically seen in athletes. The model demonstrated increased sensitivity and specificity in comparison to standard diagnostic variables (52). Valvular disease can also be assessed using AI methodology. SVM classifiers were used for classification and determination of the severity of mitral regurgitation (MR), a common valve disease. The method achieved sensitivity of 99.38% and specificity of 99.63% for the identification of the severity of MR in normal subjects (53).

The most recent advancement of AI in echocardiography concerns a video-based DL algorithm, which exceeded human experts’ performance in tasks such as EF estimation, assessment of cardiomyopathies and left ventricle segmentation. The variance in predictions from this algorithm is equivalent to or less than measurements of cardiac function by human experts. EchoNet-Dynamic is an end-to-end deep learning approach. It uses the standard apical four-chamber view echocardiogram videos as input. Spatiotemporal convolutions with residual connections are used for prediction of EF of each cardiac cycle. Weak supervision from expert human tracing is used to generate frame-level semantic segmentations of the left ventricle. The outputs are combined to create beat-to-beat predictions of the EF and the presence of HF with reduced EF (*via* AUC). EchoNet-Dynamic was created by using 10.030 apical four-chamber echocardiogram videos during training of the model. It is the first video-based DL model for echocardiogram and its performance in measuring EF is better than previous DL models. It can rapidly identify subtle changes in EF and aid the precise diagnosis of CVD in real time (54).

### Cardiac computed tomography angiography

Coronary artery disease (CAD) risk assessment is fundamental in the efforts to reduce future cardiovascular events. Traditional prediction models have limitations, including variations among the validation cohorts, a small number of predictors, and the absence of important variables. The need for robust prediction tools for accurate prediction of CAD burden and the recent advancements in AI, led to the development of ML-based risk prediction models (55). Cardiac computed tomography angiography (CTA) is a non-invasive imaging investigation that permits a direct evaluation of the patency of coronary arteries and has been vital in ascertaining the incidence of coronary artery disease (CAD) and consequent prognostication (56). Coronary artery calcium scoring (CACS) with or without CTA can provide qualitative and quantitative details on atherosclerosis, whilst CTA can determine the stenosis of an atherosclerotic lesion (32). ML methods are applied in CTAs, to maximise information extraction *via* image acquisition, and improve diagnostic accuracy and prognostic outcomes *via* precision risk stratification.

Data from 8,844 patients from a multi-centre registry were used to compare the AUC for conventional CTA risk scores in comparison to a score created using a boosted ensemble algorithm for risk stratification. With a mean follow-up time of 4.6±1.5 years, the AUC was considerably better for the ML based approach, indicating that ML can improve risk stratification, compared to the current CTA risk scores (57). In another multicentre study, 13,054 participants with suspected or previously established CAD, underwent CACS measurements. The CACS was used in a gradient boosting ML algorithm (XGBoost) (boosting tree-based ensemble algorithm), in combination with clinical risk factors, for assessment of potential improvement of risk stratification. The study showed around a 9% increase in the ability to approximate pre-test probability of obstructive CAD, when adding CACS in the baseline model. In the subgroup of younger patients (less than 65 years old) this was increased to around 17% (58). Another study investigated ML based risk stratification in an asymptomatic healthy population. 85,945 asymptomatic participants underwent a CTA scan with CACS, with 66 available parameters. A ML algorithm was used to predict moderate (CACS > 100) and high-risk (CACS > 400) CAD patients and was compared a conventional risk prediction score. 8.4 and 2.4% of the population had indication for moderate and high-risk CAD, respectively.

The study showed that the ML algorithm was superior to the conventional risk prediction score, in both the moderate and high risk for CAD groups (59). From a clinical perspective, the use of ML for prediction of CAD for all patient subgroups leads to effective precision risk stratification (exploration of all available information for calculation of each individual’s risk), less exposure radiation (as CTA with CACS has not been found to be superior of ML with CACS alone) and a more automated and accurate selection process for further diagnostic evaluation of the appropriate candidates with better clinical outcomes. The use of ML for CAD prediction aims to create risk stratification models that are more accurate and cost and time efficient in clinical practice, compared to conventional models (58).

Motwani et al., studied 10,030 patients with suspected CAD during a 5-year follow-up from an international multicentre study. All patients underwent clinically indicated CTA. 25 clinical and 44 CTA parameters were measured. The ML approach concerned automated feature selection by information ranking, model building with a boosted ensemble algorithm (LogitBoost) and 10-fold stratified cross-validation, through the whole process. The primary outcome of the study was all-cause mortality (ACM). 745 patients died during the 5-year follow up. The ML approach was identified as a significantly better predictor of a 5-year ACM, in comparison to the clinical or CTA measures alone, as indicated by the higher AUC in all comparisons (60).

Most cases of acute coronary syndrome (ACS) are caused by unstable but non-obstructive atherosclerotic plaques. The current available non-invasive diagnostic tests which detect coronary artery stenosis or stress-induced myocardial ischemia, are unable to detect these unstable non-obstructive plaques. It is established that vascular inflammation causes atherosclerotic plaque formation and rupture, leading to ACS. The perivascular fat attenuation index (FAI) is an AI-derived imaging biomarker, which captures the alterations in perivascular fat attenuation, caused by vascular inflammation. Two independent cohorts including 1,872 patients undergoing CTA, investigated the prognostic value of perivascular fat attenuation mapping for all-cause and cardiac modality. Perivascular fat attenuation mapping was performed around the three major coronary arteries. In both the derivation and validation cohorts, high perivascular FAI values around the proximal right coronary artery and left anterior descending artery, were projecting of all cause and cardiac mortality. A cut-off of −70 for the perivascular FAI was determined, above which sharp rise in cardiac mortality was observed. This ground-breaking study showed that perivascular FAI, an AI-derived biomarker, provides a quantitative measure of coronary inflammation and increases cardiac risk prediction and reclassification over current-state-of-the-art valuation *via* CTA. Non-invasive detection of coronary inflammation *via* FAI can lead to timely and aggressive initiation of primary prevention for patients with no visible CAD but unstable atherosclerotic plaques that can potentially lead to myocardial infarction if untreated. Prompt prevention will lead to reduced incidence of clinically diagnosed CAD and requirement for further intervention. FAI can also guide future trials in assessing novel but affordable therapeutic agents that target inflammation (61).

### Single-photon emission computed tomography

Assessment of myocardial perfusion correlates to the existence of obstructive CAD. This can be implemented with single-photon emission computed tomography (SPECT) stress testing (32). AI methodology has been applied in this modality, aiming to improve tasks such as image acquisition, image reconstruction and automated quantitation. An ensemble-boosting ML algorithm, LogiBoost, incorporated from clinical data and quantitative image features, compared the diagnostic accuracy of the model for estimation of obstructive CAD to the standard quantification [total perfusion deficit (TPD)] and visual analysis by two experienced readers. The accuracy of the ML algorithm was similar to Expert 1 and superior to combined supine/prone TPD and expert 2, showing an improvement in the diagnostic performance of myocardial perfusion imaging (MPI), by integration of ML (62). The automatic prediction of obstructive CAD from myocardial perfusion imaging (MPI) by DL, compared to TPD, was assessed in a more recent multicentre study. 1,638 patients without known CAD underwent stress MPI and invasive coronary angiography (ICA) within 6 months of MPI. AUC for disease prediction (obstructive disease defined as > 70% of narrowing of coronary arteries) by DL was higher than TPD, showing the potential for improvement of automatic interpretation of MPI, by AI methods (63). In another multicentre study, 1,980 patients with suspected CAD, underwent stress MPI with novel SPECT scanners. All patients had subsequent ICA within 6 months. LogiBoost, was also utilised in this study to forecast early coronary revascularisation within 90 days after the SPECT MPI and was compared to standard quantitative analysis (TPD) and expert interpretation. The ML outperformed both TPD and expert analysis, with an AUC of 0.79 (64).

### Cardiac magnetic resonance imaging

Cardiac MR is an imaging modality utilised for non-invasive assessment of CVD. It evaluates the cardiac morphology, function, perfusion, and quantitative myocardial tissue measurement (65). It is considered the gold standard for non-invasive evaluation of EF and left ventricular volume (66). CMR is broadly used for the diagnosis of cardiomyopathies, congenital heart disease, valvular heart disease, IHD, pericardial lesions, and cardiac tumours. However, it requires acquiring images characterised by high temporal and spatial resolution, different contrasts and/or whole-heart coverage, leading to a lengthy scanning time. ML incorporation to CMR, can lead to a more efficient scanning and accurate interpretation process. DL based MRI reconstruction is based on a model which learns the factors of the reconstruction procedure beforehand, so that it can be applied to all new data as a simple operation. Most important tasks utilised with DL are image construction, image segmentation and image quality control in the field of CMR (65).

Whilst CMR is executed at high resolution, analysis of the scan by the clinician remains variable, time consuming and prone to errors. Deep learning methodology has been used to overcome the challenge of automated derivation of information from CMR images (67). An automated 2-dimensional CNN (adapted from the VGG-16 network) was used to take CMR image as input, learn image features from fine to coarse scales through a series of convolutions, concatenate multi-scale features and predict a pixelwise image segmentation (67). The CNN was trained on 599 independent multicentre disease cases and subsequently was compared to an expert cardiologist and a trained junior cardiologist for the identification of left ventricular chamber volumes, mass, and EF, from 110 patients who underwent scan: rescan CMR within a week. The study showed that clinicians can detect a 9% change in LVEF with the greatest source of human error being attributed to the observer. The precision of the CNN was similar to human analysis, but its performance was 186 times faster (68). In another study, a ML approach was used for the identification of diagnostic features in pulmonary arterial hypertension (PAH) using CMR. A multilinear principal component analysis (MPCA) algorithm was utilised to extract low-dimensional features from high dimensional input to tensor representations of data. The algorithm distinguished patients with and without PAH with higher accuracy in comparison to manually drawn CMR measurements with an AUC of 0.92. Additionally, the diagnosis that used the ML approach was less time consuming (within 10 s) and had less variability (69).

Bai et al., trained a 16-layer CNN (adapted from the VGG-16 network) on a 4,875-subject dataset from the UK biobank, to automatically analyse CMR images. Its performance was assessed using technical [dice coefficient (metric of the similarity of the two segmentations)] and clinical [left ventricle (LV) end-diastolic volume (LVEDV) and end-systolic volume (LVESV), LV mass (LVM); right ventricle (RV) end-diastolic volume (RVEDV) and end-systolic volume (RVESV)] parameters. The automated method achieved great performance in segmentation of the LV and RV on short-axis CMR images (dice metric of 0.94 and 0.90 accordingly) and the left atrium (LA) and right atrium (RA) on long-axis images (dice metric of 0.93 and 0.96 accordingly), from an intra-domain UK biobank test set of 600 subjects. The automated method using the CNN was comparable to human inter-observer variability (67). CNNs perform segmentation tasks with great precision if the training and test images originate from the same scanner or site. However, their performance decreases when the test images come from different scanners or sites. Chen et al., trained a 2D CNN (U-net) on 3,975 subjects from the UK biobank. The U-net architecture has been the most widely used architecture for biomedical segmentation. In Chen’s paper, the network was identical to the original except of two differences, application of batch normalisation after each hidden convolutional layer to stabilise the training and the use of dropout regularisation after each concatenating operation to avoid over-fitting. The U-net was tested on 600 subjects from the UK biobank for the purpose of intra-domain testing and on 699 subjects from two other sets (ACDC dataset and BSCMR-AS dataset) for cross-domain testing. Chen’s proposed method was compared to Bai’s automated methodology. Despite both achieving comparable dice scores on the intra-domain UK biobank test set with high accuracy, Chen’s method achieved superior mean dice scores for all of the three structures (LV, RV myocardium) on the two cross-domain datasets. The proposed method achieved better overall segmentation accuracy with lower variance on the three datasets, improving CNN-based model generalisability for the CMR image segmentation task across different scanners and sites (70).

Late gadolinium enhancement (LGE) CMR imaging is the cornerstone of non-invasive myocardial tissue characterisation. An important example consists of the relationship between the presence and extend of LGE and adverse outcomes, in patients with HCM. However, LGE necessitates the administration of an intravenous gadolinium-based contrast agent, which should be used cautiously in patients with severe renal failure or allergy to gadolinium-based contrast. In a recent RCT of 1,348 patients with HCM, a new DL driven CMR technology named virtual native enhancement (VNE), was used to generate images identical to the standard LGE, without the need of a gadolinium-based contrast agent. The DL algorithm consisted of three parallel CNNs streams which processed and enhanced signals in native T1 maps (pixel-wise maps of tissue T1 relaxation times) and cine imaging (sequence of images at different cardiac phases) of cardiac structure and function. Each stream had an encoder-decoder U-net architecture. The encoder computed image features from fine to coarse and produced a multiscale feature representation, which the decoder combined to produce final feature maps. The feature maps from U-nets were concatenated and input into a further neural network, to produce a final VGE image. The neural networks were trained with the use of a modified conditional GAN approach. When compared, the VNE imaging achieved better image quality than LGE and was in high agreement with it in visuospatial distribution and myocardial lesion quantification. Overall, VNE resembles conventional LGE, but does not require intravenous access or administration of contrast, can be repeated if required to confirm the imaging findings without the consequences of giving contrast, and can be completed within 15 min as uses native imaging. Its advantages make it an attractive technology which can potentially be extended for diagnosis of other myocardial pathologies in the future (71).

In addition to image construction and segmentation, DL methodology has recently been utilised for image quality control purposes in the field of CMR. In a retrospective study of 3,827 subjects (including healthy and pathological hearts), a set of algorithms including CNNs were used for the development of a framework for the automatic detection and quality-controlled selection of cine images, used for cardiac function analysis from routine clinical CMR exams. The framework encompassed three steps. The first pre-processing step excluded still images. In the second step, one CNN classified images in standard cine views and a second CNN classified images depending on the image quality and orientation. The final algorithm selected one good image of each class, which was then used for analysis of cardiac function. The classification CNN achieved accuracy between 0.989 and 0.998, whilst the quality control CNN achieved accuracies of 0.861 for 2-chamber, 0.806 for 3-chamber, and 0.859 for 4-chamber views. The complete framework also achieved accuracies of 89.7, 93.2, and 93.9% for 2-, 3-, and 4-chamber acquisition from each study, respectively. This study demonstrates the future potential for high quality automated cine CMR analysis from the scanner to report (72).

### Heart failure

Heart failure (HF) affects 1–2% of the adult population in developed countries and more than 10% of patients > 70 years of age (73). Early diagnosis or prediction of HF has a high impact on successful treatment and prolongation of life expectancy for patients. ML applications have been used for the early detection of HF, classification, severity estimation, and prediction of adverse events (e.g., 30-day re-hospitalisation) (32).

A multi-level risk assessment for developing HF, with prediction of five risk levels (no risk, low, moderate, high, and extremely high risk), *via* the use of a decision tree classifier, was established in a study by Aljaaf et al. They also added three new risk factors (obesity, physical activity, and smoking) in a previously used dataset and enhanced the accuracy of predicting HF. This was the first study with a multi-level prediction of HF, in contrast to the binary outcomes from previous studies. The predictive model showed an improvement from existing studies with a sensitivity of 86.5% and specificity of 95.5% (74) A SVM was trained on clinical parameters from 289 patients and triaged patients into three categories (HF, HF-prone, and healthy). The overall classification accuracy was 74.4%, with precisions of 78.79, 87.5, and 65.85% for ascertaining the healthy group, HF-prone group and HF group, respectively. The scoring model showed improved accuracy in classification of HF, in comparison to clinical practice criteria (25–50% accuracy) (75).

More recently, a DL approach was used to screen individuals for asymptomatic left ventricular dysfunction (ALVD). ALVD is prevalent in 1.4–2.2% of the population and if left undiagnosed, it can lead to increased morbidity and mortality. Early identification of ALVD and commencement of treatment, can prevent its progression to symptomatic HF and reduce mortality. A CNN was trained on 12-lead-ECG and echocardiography information, including LVEF, from 44.959 individuals to identify patients with ventricular dysfunction (EF 35%). The CNN was composed of three parts. Six convolutional blocks (convolution, batch normalisation, Relu, max pooling) extracted temporal features, one convolutional block (convolution, batch normalisation, Relu) extracted spatial features and two fully connected layers (fully connected, batch normalisation, Relu, dropout) regressed the features to a softmax activated output. The DL algorithm was tested on a set of 52.870 patients and showed AUC, sensitivity, specificity and accuracy of 0.93, 86.3, 85.7, and 85.7%, respectively. Patients without ventricular dysfunction with a positive AI screen were four times more probable to develop future ventricular dysfunction, in comparison to patients with a negative screen. An inexpensive, non-invasive test such as AI screening from ECG data, can be a powerful future tool for screening asymptomatic individuals (76).

Readmission rates after hospitalisation with HF remain high and lead to increased disease burden and costs for healthcare systems. The performance of current predictive methods for the likelihood of HF readmissions is modest. Accumulating publications show positive results from ML-driven methods for predicting readmission of patients with HF (77–79). In one of the studies, EHRs were used to enrol 1,653 patients within 30 days of their discharge after an index admission for HF. ML algorithms were compared to the traditional method of logistic regression (LR), for effectiveness in predicting 30 and 180 days all cause readmissions and readmissions due to HF. For the 30-day all-cause readmission prediction, random forest (RF) showed a 17.8% improvement over LR. For readmissions because of HF, boosting showed a 24.9% improvement over LR. Lastly, the ML models stipulated enhanced recognition of groups at low and high risk for readmission, by increasing the predictive range compared with LR (80). A retrospective study applied DL methodology [deep unified networks (DUNs)] to data from EHRs of 11,510 patients, in order to generate a risk prediction model to forecast 30-day readmissions in patients with HF. DUNs consist of a mesh-like network structure which avoid overfitting. The DUNs’ AUC (0.705) had the best result of 10-fold cross-validation, compared to LR (0.664), gradient boosting (0.650) and maxout networks (activation function used in neural networks) (0.695). The DUNs model also showed an accuracy of 76.4% at the classification threshold with greatest net savings for the hospital (81).

Cardiac resynchronisation therapy (CRT) is fundamental to the management of symptomatic HF with left ventricular systolic dysfunction and intraventricular conduction delay (reduced EF and wide QRS complex). Conventionally, patients eligible for CRT implantation, should have an ECG morphology with LBBB and QRS duration ≥ 150 ms. Patients with these ECG characteristics have greater benefit on reduction of mortality and readmissions after receiving CRT. However, around 30% of patients meeting these criteria and receiving an implant, do not experience clinical benefit from CRT. Therefore, predicting a patient’s outcome after CRT is an essential step in the decision-making procedure pre-implantation. In a retrospective study, ML models were developed for prediction of ACM or HF hospitalisation at 12 months post-CRT. Clinical characteristics and ECG features were used in model development. A RF algorithm was found to be the best performing model, when compared to other ML models and the traditional ECG prediction methodology (LBBB and QRS ≥ 150 ms). Whilst the ECG morphology did not reach significance for differentiation of survival difference across subgroups (*p* = 0.08), the RF model formed quartiles of patients with an 8-fold change in survival between those with the maximum and minimum predicted probability for events (*p* < 0.0001). Furthermore, the RF model achieved better discrimination of the risk of the composite end point of ACM and HF readmission, than the ECG morphology-based subgroup analysis (82).

Another retrospective study concerned data from 1,510 patients who underwent CRT implantation. ML models were trained from 33 pre-implant clinical features, to predict 1–5-year ACM. The best performing ML model (highest AUC for the prediction of all-cause mortality at 1, 2, 3, 4, and 5 year follow up), a RF model, was chosen for further assessment and it was referred as the SEMMELWEIS-CRT score. This was compared to pre-existing scores and showed significantly better response prediction and improved discrimination of mortality. An online calculator was developed, which will enable a personalised calculation of predicted mortality in patients undergoing CRT implantation (83). Similarly, in another study a ML-based approach was used to phenogroup a HF cohort and identify responders to CRT. 1,106 patients from a multicentre trial, were randomised into two groups [CRT with a defibrillator (CRT-D) or an implantable cardioverter defibrillator (ICD)]. An unsupervised ML algorithm, *via* dimensionality reduction and clustering, classified patients into groups, based on clinical parameters, left ventricular volume, and deformation traces at baseline. The treatment effect of CRT-D on the primary outcome (all cause death/HF event and on volume response) was compared among the different groups. From the four phenogroups identified, two had a greater proportion of known clinical characteristics prognostic of CRT response and were linked to an improved treatment effect of CRT-D on the primary outcome (84).

Machine learning has enabled physicians to use data from ECGs and draw specific echocardiography results, without the use of the echocardiogram. A randomised controlled trial (RCT) aimed to identify patients with low ejection fraction (EF), *via* AI-enabled ECGs. Low EF is an important marker of heart failure which can be effectively treated to improve survival if recognised early. The study showed that the use of an AI algorithm (using neural networks) based on ECGs, led to the diagnosis of patients with low EF at an early stage in the setting of routine primary care (85). Other studies enabled the calculation of other parameters such as left ventricular hypertrophy (LVH) and left ventricular diastolic function (LVDF) based on ECG features and ML methods (86, 87).

Machine learning application in the field of HF, has enabled the accurate prediction of HF, ALVD, and low EF in asymptomatic individuals. Early identification of patients at risk of developing HF or at an early onset of the condition, can lead to prompt and aggressive primary prevention/initiation of treatment and more rigorous follow up of these patients, with improved clinical outcomes. Prompt detection of patients who might require re-admission after hospitalisation with HF, can initiate a well-structured outpatient pathway for this cohort, which will aim to keep these patients in the community and reduce re-admissions and subsequent morbidity and mortality and hospital costs. This could be achieved, through liaison of the secondary care team with the community heart failure team and the patients’ GP and maximisation of their treatment in the community, *via* more regular reviews at home, at the GP practice or in an outpatient clinic or ambulatory setting. Moreover, accurate and personalised prediction of the cohort of patients which would have a good outcome if having a CRT-D inserted, can lead to the reduction of unnecessary procedures (and subsequently reduced hospital costs and resources) and the associate medical risks for those patients who would not have the same outcome. Overall, the incorporation of ML methodology into the field of HF aims the early detection of those patients most at risk of developing the disease, correct classification of patients based on their personalised risk and prompt intervention which can be beneficial for both the patients (improved morbidity and mortality *via* early initiation of treatment) and secondary care (*via* shifting treatment and follow up in the community and reducing hospital admissions). Lastly, an important aspect of ML models and their application in clinical practice is the myriad of signals they can highlight within the data, which can potentially aid in the better understanding of a particular aspect of the disease (which would not be noticeable either way) and lead to further scientific discoveries in the future.

### Other applications

An algorithm for heart murmur detection was developed in a virtual clinical trial, with aim to enhance the precision of screening for valvular and congenital heart diseases. 3,180 heart sounds recordings (pathologic murmur, innocent murmur, no murmur), from 603 outpatient visits were chosen from a large database. Algorithm assessment of heart rate (HR) showed great similarity to the gold standard. Pathologic cases were identified with sensitivity of 93%, specificity of 81%, and accuracy of 88%. This trial was the first to objectively evaluate an AI-based murmur detection algorithm, making it a potentially useful screening tool for heart disease (88).

Various studies have been carried out to demonstrate effectiveness of AI-driven phenogrouping, in all fields of cardiology. Such landmark study concerns the development of a phenomapping-derived tool, for selection of anatomical or functional testing in patients with stable chest pain. The decision support tool named ASSIST (Anatomical vs. Stress testing decision Support Tool), was developed using data from the PROMISE (PROspective Multicentre Imaging Study for Evaluation of Chest pain) trial. Data from 9,572 patients undergoing anatomical (4,734) or functional (4,838) imaging were used to create a topological presentation of the study populations, based on 57 pre-randomisation variables. Individual patient-centred hazard ratios for MACE were calculated with Cox regression models, within each patient’s 5% topological neighbourhood, leading to heterogeneity in the map and distinction of phenotypic neighbourhoods favouring either anatomical or functional imaging. A gradient boosting algorithm was used in 80% of the PROMISE population, in order to predict a personalised outcome if using anatomical or functional testing and create the ASSIST tool. The ASSIST tool was tested in the rest 20% of the PROMISE population and in an external validation cohort (from the SCOT-HEART trial), undergoing anatomical or functional testing as first assessment. The testing stagey recommended by ASSIST showed a significantly lower incidence of each study’s endpoint and of ACM or non-fatal AMI. The personalised novel tool can support physicians in the decision to proceed with anatomical or functional testing when evaluating patients with stable chest pain (89).

An interesting example of the use of AI methodology, is a recent study by a Chinese group of scientists who developed a CNN (50-layer ResNet classification network) which detected CAD (stenosis > 50% documented by angiography), *via* analysing the patient’s facial photo. 5,796 patients were divided to a training (5,216) and validation sets (580) for the algorithm development. The AI algorithm’s AUC was 0.730 and was found to be higher than the standard prediction scores. Sensitivity was 80% and specificity was 54%. Further studies would need to be conducted, as the study had several limitations including the geographical characteristic of the cohort (only Chinese population). Significant CAD was defined based on coronary angiogram or CTA data, which led to small selection bias, which could have potentially altered the algorithm outcome. Lastly, in the visualisation tests, the cheek, forehead, and nose contributed more to the algorithm than other areas of the face. This could be the result of the extraction of features by the DL algorithm that are associated with CAD but are not obvious to human observers. Despite its limitations, this example shows the vast advancements and the future potential of AI applications in cardiology, with the generation of results from a simple intervention such as taking a selfie (90)!

In a recent prospective, single-centre study, contactless facial video recording was used to train a 12-layer DNN for the detection of asymptomatic AF. A camera based remote photoplethysmography (rPPG) was used during a 10-min facial video recording of 453 study participants. Its signals were extracted and segmented into 30 s clips, which were used to train a CNN. If more than 50% of the subject’s rPPG segments were identified as AF rhythm by the model, the participant would be classified as AF. The accuracy of the DL model for discrimination of AF from NSR and other ECG abnormalities, was compared to the standard 12 lead ECG. The DL model achieved a 90 and 97.1% accuracy in detecting AF in 30 s and 10-min recordings, respectively (91).

### Internet of things

Internet of Things (IoT) is described as “*a network of devices interacting with each other via machine to machine (M2M) communications, enabling collection and exchange of data*” (92). IoT can be applied in medicine in fields such as remote health monitoring, chronic diseases management, obedience to treatment and medication at home and elderly care (93). When it comes to cardiology applications, IoT can be applied for the identification of cardiac emergencies remotely. Wolgast et al., designed a body area network for measurement of an ECG signal and its transmission *via* Bluetooth to a smartphone for data analysis. The user’s own smartphone would process the data and built-in communications could be used to raise an alarm if a heart attack was identified (94). In another study, subjects were observed for a period of 3 months, using a wearable sensor which documented physiological data. Data were uploaded constantly *via* a smartphone to a cloud analytics platform. A ML model was used to design a prognostic algorithm which detected HF exacerbation and predicted rehospitalisation after a HF admission (95).

## Ethical dilemmas

Despite its huge potential, AI is still something new, unfamiliar, and sometimes difficult to comprehend. It therefore carries various ethical dilemmas and limitations that need to be addressed. Firstly, the design of studies based in AI and the training and validation process of the new technology, can be flawed. Most of the studies reporting AI applications have retrospective design and small sample size, which can potentially lead to bias. More importantly, AI-driven studies can have selection bias, which includes sampling and observer selection bias (32). From another perspective, since AI-driven technologies achieve their results from existing features and dynamics of the populations they analyse, this can lead to reproduction, amplification of patterns of marginalisation, inequalities and discrimination that exists in these populations. Again, the features of the data chosen to train the algorithms are chosen by the investigators and the AI-driven application can replicate the investigator’s preconceptions and biases (96).

Foundation models are AI systems that are trained on broad data and can be adapted to a variety of downstream tasks (33). An example of such model is AlphaFold, which is an AI system developed by DeepMind and can predict a protein’s 3D structure from its amino acid sequence. These predictions are easily available to the scientific community and can provide individual downloads for the human proteome and the proteomes of 47 other key organisms in research (97). Such systems require vast amount of data for training and large amount of computing resources to effectively use that data. Consequently, the ownership of the data and their models is often centralised, giving power and decision rights to organisations with the most resources (big tech companies) and reducing opportunities for others, leading to inequalities, or one can say anti-democratic situations. Despite efforts to build models through distributed training, it is highly likely that this large gap will remain between the two (98).

Since ML models learn on high dimensional correlations which exceed the interpretive abilities of humans, the rationale behind algorithmically produced outcomes which affect decision making for patients, can remain unjustifiable. The absence of a familiar logic behind its output, might lead the clinician who is interpreting it to pause. Also, when decisions, predictions or classifications are made based on AI systems, individuals are unable to hold direct accountability for these outcomes. In the case of harm of the patients, this accountability gap can affect their autonomy and violate their rights (96). The question of trusting a system, which might not even be understood by the decision maker, is raised. In the end, could such systems beat a clinician’s judgement, if there is a conflict in management plans? For example, would the automated diagnosis of an ECG-reading algorithm saying that a patient has a STEMI (ST elevation myocardial infarction) surpass a clinician’s view who is aware that the ECG changes are due to long-standing LBBB? The answer is no. Recently, it was found that DL models made incorrect decisions by using cautiously engineered inputs, raising concerns that such systems are not yet ready for mainstream use (99). The same concern is raised with non-robust CNNs and ML models under various circumstances, such as in the previously discussed case of adversarial attacks. Physicians should have the last and most important say when AI is applied for decision-making in the medical field.

Another recent development, which aims to mitigate the famous issue of “black-box” AI methodologies, is explainable AI (XAI). As AI becomes more advanced, it is less understood by humans. Whilst lower performance systems such as ML learning are more understandable, higher performance models such as DL techniques are difficult to comprehend even from the engineers or data scientists who created the algorithms, since they are directly created from data. In safety critical situations, such as in medicine, the non-transparency of these techniques can lead to wrong decision making and pose serious danger to human lives (100). The aim of XAI is to allow humans to comprehend how the algorithm works, trust its results and the output it produces, unmask potential biases, and characterise the model’s accuracy and transparency. XAI ensures AI systems meet regulatory standards and adopt good practice towards accountability, making them easier and faster to deploy in businesses and high risk environments such as the medical field (101).

The rapidly increasing use of smart medical devices and digital health applications through IoT and AI, imposes a danger of dehumanisation of medicine. More and more intelligent applications replace the work of physicians in various sectors. For example, the detection of new AF *via* a wearable device, aids the diagnosis of a potential arrythmia, but takes away the clinician’s opportunity to demonstrate their knowledge and practice their skills (e.g., detect the arrythmia from a real time ECG for the first time). On the other hand, the potential of AI in reducing admin burden for physicians (e.g., analysing EHRs), can create the opportunity for having more interaction and quality time with their patients. Balance is what is required, in order to maintain healthy physician to patient relationship, with integration of AI technologies when needed to relieve admin burden (102).

Introduction of IoT and AI-driven tools for medical monitoring of various parameters in individuals has generated ethical concerns (102). AI technologies can utilise such personal data, without obtaining the proper consent of the data subject or handle it in a manner personal information is revealed. One can say that the ability to lead a private life, could be jeopardised (96). The concern of personal data privacy is raised, as most data protection laws are based on principles established in 1980, which might not be reflecting the current reality. As per current laws, personal data should be collected and used for a specific purpose. Also, data should be sufficient, relevant, and restricted to what is required in the context of the purpose of its use. However, when AI is concerned, neither of the above can be guaranteed. The AI algorithms are complex, not always understood by their programmer, can generate surprisingly different results from what was expected and can lead to a change in the purpose, through the learning and development process. Data can neither be restricted or deleted after its original use, as keeping data is vital for the models’ optimal performance. It is now more essential than ever that the data protection laws are re-visited and adjusted to work better towards data privacy issues arising from the vastly growing fields of AI, big data and IoT (103).

## Translation of artificial intelligence to future clinical practice

Despite the landmark studies exhibiting the potential of AI in transforming medicine, the ethical dilemmas concerning its real-life implementation are still unaddressed. AI systems can be flawed and their generalisability to new populations and settings, may produce bad outcomes and lead to poor decision-making.

Going forwards, education of scientists, physicians but also of the public regarding AI and the logic behind its applications is vital. This can lead to better understanding and improved engagement in commercialisation of AI applications. Medical engineering has been incorporated in several universities’ curriculum. Subjects such as computational sciences, coding, and algorithmics, should also be incorporated in the curriculum. Universities have also started providing short courses and postgraduate level degrees on AI in healthcare. Educated physicians in AI, could aid adoption of innovative applications, but also raise awareness when ethical and privacy issues are risen.

Another important aspect is the achievement of robust regulation and quality control of AI systems. As AI is a new and rapidly evolving innovative field, it carries significant risks if underperforming and unregulated. As previously mentioned, the FDA has recently released a regulatory framework with aim to establish safe and effective AI- based medical devices, which can progress for patient use (104). The European Union has also proposed a regulatory framework on the use of AI, with plan to come into force in the second half of 2022, in a transitional period (105). Regulations should also be established for upgrades of AI products, throughout the lifespan of the product. Some AI systems have been built with continuous updates, but this could potentially result to drift with time. Periodical updates after a complete evaluation of the clinical significance of the AI product are preferred. Guidelines should also be developed for the purpose of evaluation of the product’s performance and the detection of deficits over time (106).

Due to the various limitations and ethical dilemmas AI carries and its potential harm to the public, it is necessary to incorporate AI ethics and safety, during the development of AI systems. AI ethics “*is a set of values, principles, and techniques that employ widely accepted standards of right and wrong to guide moral conduct in the development and use of AI technologies*.” An ethical platform is required for the responsible delivery of an AI project. This necessitates cooperation from all the team members of the multidisciplinary team, in order to maintain a culture of responsibility and execute a governance architecture that will adopt ethically practices at every point in the innovation and implementation lifecycle. Overall, the AI project needs to be ethically acceptable, fair and non-discriminatory, justifiable and worthy of public trust (96).

## Conclusion

In our fast-paced world, time is precious and limited. Healthcare is facing a crisis of understaffed departments and more informed patients who demand the best treatment. There is an unmet need for the effective triage of patients, efficient clinical evaluation and incorporation of clinical expertise with evidence-based medicine and the latest technologies and accurate decision making for the right diagnostics and treatment plans. AI will be a part of every cardiologist’s daily routine to provide the opportunity for effective phenotyping of patients and design of predictive models for different diseases. It will enhance the use of non-invasive diagnostics and reduce the need for costly and complicated invasive tests, for the diagnosis of CAD. Future cardiologists will be able to tell an asymptomatic patient, whether they will develop a lethal arrythmia or an MI and what needs to be done to avoid this. Cardiologists should educate themselves in the development of AI and take part in AI innovations and utilise them in their practice. However, they will need to take into consideration the ethical dilemmas generated in areas where AI is replacing human and aim to integrate their knowledge and AI-derived suggestions, for a mature and accurate decision making in every step in the decision process.

## Author contributions

All authors listed have made a substantial, direct, and intellectual contribution to the work, and approved it for publication.

## Glossary

ACM: all-cause mortality
ACS: acute coronary syndrome
AI: artificial intelligence
AF: atrial fibrillation
AHA: American heart association
ALVD: asymptomatic left ventricular dysfunction
AMI: acute myocardial infarction
AUC: area under the curve
CACS: coronary artery calcium scoring
CAD: coronary artery disease
CNN: convolutional neural network
CRT: cardiac resynchronisation therapy
CT: computerised tomography
CTA: cardiac computed tomography angiography
CTP: computed tomography myocardial perfusion
CVD: cardiovascular disease
DD: diastolic dysfunction
DL: deep learning
DUN: deep unified network
ECG: electrocardiogram
ECR: early coronary revascularisation
EF: ejection fraction
EHR: electronic health record
ESC: European society cardiology
FAI: fat attenuation index
FDA: food and drug administration
FNNN: feed forward neural network
HCM: hypertrophic cardiomyopathy
HF: heart failure
HFpEF: heart failure with preserved ejection fraction
HR: heart rate
ICA: invasive coronary angiography
IoT: internet of things
LBBB: left bundle branch block
LR: logistic regression
LS: longitudinal strain
LV: left ventricle
LVDF: Left ventricular diastolic function
LVEDV: left ventricle end diastolic volume
LVEF: left ventricular ejection fraction
LVESV: left ventricle end systolic volume
LVH: left ventricular hypertrophy
LVM: left ventricle mass
MACE: major adverse cardiac event
ML: machine learning
MLP: multiplayer perceptron
MNN: modular neural network
MPI: myocardial perfusion imaging
MR: mitral regurgitation
PAH: pulmonary arterial hypertension
PCI: percutaneous coronary intervention
PVC: premature ventricular contraction
RBBB: right bundle branch block
RBFN: radial basis function network
RCT: randomised controlled trial
RF: random forests
RNN: recurrent neural network
ROC: receiver operator characteristic
RV: right ventricle
RVEDV: right ventricle end diastolic volume
RVESV: right ventricle end systolic volume
SPECT: single-photon emission computed tomography
SR: sinus rhythm
SVM: support vector machine
TPD: total perfusion deficit.
